# Children With Special Health Care Needs: Acknowledging the Dilemma of Difference in Policy Responses to Obesity

**Published:** 2011-08-15

**Authors:** Paula M. Minihan, Aviva Must, Betsy Anderson, Barbara Popper, Beth Dworetzky

**Affiliations:** Department of Public Health and Community Medicine, Tufts University School of Medicine; Tufts University School of Medicine, Boston, Massachusetts; Family Voices, Boston, Massachusetts; Federation for Children With Special Needs, Boston, Massachusetts; Mass Family Voices at the Federation for Children With Special Needs, Boston, Massachusetts

## Abstract

Children with special health care needs (SHCN) account for part of the increasing prevalence of childhood obesity in the general population and can face an elevated risk for obesity. The federal government, in partnership with states, has assumed the role of steward for this vulnerable population and supports a network of services designed to promote their health through increased access to quality health services. Addressing obesity-related health risks among children with SHCN requires policies that support family- and community-based initiatives in addition to health services. We discuss the ethics of child obesity policy from the perspective of children with SHCN and their families, and identify salient issues to optimize benefits for children and families. We refer to the dilemma of difference to identify policy concerns that are specific to children with SHCN and ethically may require different approaches. Determining the appropriate mix of inclusive and special obesity prevention initiatives for children with SHCN and identifying approaches to ensure their full participation in community-based obesity prevention activities present challenges. Children with SHCN from low-income and minority communities are particularly vulnerable and warrant special attention.

## Introduction

During 2008, approximately 1 in 7 children younger than 18 years (10.2 million children) were classified as having special health care needs (SHCN) according to the federal definition (1). Estimates of the prevalence of overweight and obesity among children with SHCN obtained from nationally representative surveys revealed that they contribute to the increase in childhood obesity in the general population (2-4). Advances in medical care and successes of policy initiatives have improved children's access to quality health services, and the majority of children with SHCN now live close to normal lifespans in relatively good health (1). Childhood obesity, which is associated with health problems throughout life, is a particular threat to children with SHCN and may slow or reverse other health gains. Obesity is a stigmatizing condition and can be another characteristic that identifies these children as different (5). Interventions for childhood obesity have not yet been included in the formal support and policy networks designated for children with SHCN. Multifactorial efforts to promote healthy weights in children do not routinely include children with SHCN, according to anecdotal information. Policy responses to childhood obesity must reach children with SHCN, yet ethical concerns about child obesity policy may thwart development of workable solutions (6). In this article, we discuss the ethics of child obesity policy from the perspective of children with SHCN and their families. Our goal is to identify the issues that are most pertinent to efforts to optimize benefits of child obesity policy for children with SHCN. We describe the profile of these children and review what is known about the prevalence of overweight, obesity, and associated risk factors to inform the policy discussion.

## Overweight and Obesity Among Children With Special Health Care Needs

### Definition of special health care needs

Children with SHCN constitute a heterogenous group linked both by the presence of a chronic physical, developmental, behavioral, or emotional condition and the need for health and related services that differ from those required by children generally in type and in intensity (7). The federal Maternal and Child Health Bureau (MCHB) promulgated this definition to help states develop coordinated systems of care to address the complex and specialized needs of families raising children with chronic medical and behavioral conditions (7). National estimates of children with SHCN are derived primarily from random-digit–dialed population-based household telephone surveys that use the MCHB definition. This article draws from 2 surveys: the National Survey of Children with Special Health Care Needs (CSHCN) 2005-2006 (1) and the National Survey of Children's Health (NSCH) 2007 (2). Both surveys use the CSHCN Screener, a validated screening instrument for identifying children with SHCN as defined by MCHB. When households with children younger than 18 years are identified, all children younger than 18 are screened as follows: parents who identify a child as having any of 5 different health consequences are asked if it is due to a medical or behavioral condition of at least 1 year's duration. Only NSCH 2007 (2) includes data to characterize weight status or obesity.

### Prevalence of special health care needs

Prevalence varies by sex, age, and race/ethnicity, but not by income (Table 1) (1). Prevalence is higher among males and increases with age. It is highest among children identified as multiracial, followed by children who are white, black, and American Indian/Alaska Native; it is lowest among children who are Hispanic and Asian. Approximately three-fourths of children with SHCN (78.4%) need or use prescription medication. Almost 4 of 10 (38.5%) need or use more medical care, mental health, or educational services than children without SHCN (Table 2). Allergies (53.0%), asthma (38.8%), and attention deficit disorder (ADD)/attention deficit hyperactivity disorder (ADHD) (29.8%) are the most prevalent conditions.

### Estimates of overweight and obesity

NSCH (2), the first nationally representative survey to query the weight status of children with SHCN using the MCHB definition, estimated that 36.3% of children aged 10 to 17 years with SHCN were overweight or obese (defined as having a body mass index [BMI] >85th percentile for age and sex), compared with 30.2% of children developing typically. Children aged 10 to 17 years with select chronic physical, developmental and behavioral or emotional conditions were at increased risk for obesity (defined as BMI >95th percentile for age and sex) compared to children without a chronic condition, according to analyses of NSCH 2003 data that controlled for socioeconomic risk factors (4). Obesity estimates from NSCH are based on parent-reported measures of weight and height. Estimates from the National Health and Nutrition Examination Survey 1999-2002, based on direct measures of weight and height using a standard protocol, also indicate a higher prevalence of obesity among children with developmental disorders compared to children without such conditions (3). Estimates derived from smaller nonrepresentative samples also suggest that children with SHCN have an elevated risk for obesity (8-10).

### Risk factors for obesity

Obesogenic environments, characterized by unhealthy food choices, limited opportunities for physical activity, and many opportunities to be sedentary, challenge most US families and children, including children with SHCN (11). Some children with SHCN represent racial/ethnic minorities or come from low-income families, factors that contribute to increased risk (1). Children with SHCN also face risk factors that are uniquely related to their special needs (12). Certain obesity risk factors are associated with underlying conditions (eg, Prader-Willi syndrome). More commonly, risk factors are secondary to underlying conditions. Children with SHCN, for example, may have less healthy dietary and physical activity patterns because of medical conditions (eg, spina bifida or cerebral palsy) that limit or restrict opportunities to be physically active (13,14). According to nationally representative data, fewer children with SHCN (aged 6-17 y) are engaged in recommended vigorous physical activity at least 4 days per week compared to children without SHCN (60.9% vs 65.3%, respectively) (2), and more children with SHCN (aged 6-17 y) watch television or videos or play video games at least 4 hours per weekday compared to children without SHCN (12.8% vs 10.3%, respectively) (2). No nationally representative data exist regarding the degree to which children with SHCN meet national dietary guidelines, although studies report dietary deficiencies (13-15).

Medication-induced weight gain may partially explain the higher obesity prevalence among children with SHCN, although it is believed to be responsible for only a limited proportion of childhood obesity nationwide. Approximately 8 of 10 children with SHCN take at least 1 prescription drug (1). Thirty percent of children with SHCN have ADD/ADHD and 21% have depression, anxiety, or other emotional problems (1), conditions that are sometimes managed by medications associated with weight gain. Medications associated with weight gain in children include the atypical antipsychotic medications (eg, risperidone), antidepressants, mood stabilizers, and anticonvulsants (eg, valproate) (16,17).

## The Ethics of Child Obesity Policy

We discuss 4 ethical considerations from the perspective of children with SHCN and identify salient issues: 1) competing political perspectives on child obesity policy, 2) the stewardship model, 3) the dilemma of difference, and 4) ethics and the role of families in policy making.

### Competing political perspectives on child obesity policy

Competing political perspectives figure into the discussion of obesity (6). Proponents of one perspective view obesity as a personal responsibility. They typically oppose policy responses to obesity and view them as unethical because they interfere with personal autonomy. Proponents of a competing perspective see obesity as a consequence of obesogenic environments amenable to government intervention.

Children with SHCN and their families have a policy history that reflects both perspectives. Families have always assumed major personal responsibilities for the health of children with SHCN above those required of other families. Most families acknowledge that government has been instrumental in expanding opportunities for children with SHCN and improving their quality of life. Families of children with SHCN are heterogenous, and their responses to obesity prevention initiatives vary. Families that view childhood obesity as requiring solutions involving both personal responsibility and government involvement see themselves as having an important role in encouraging their children to eat well and be physically active (18). They are also open to policies designed to help all families promote healthy weights. Families appreciate mainstream initiatives that allow their children to benefit from being like their peers, but they are also aware that their children might not be able to participate fully in mainstream activities. Efforts must be made to assure families that their children will be included and welcomed in community initiatives. The policy history for these families probably will encourage support for child obesity policy; meanwhile, the dilemma of difference influences which approaches families deem feasible or desirable.

### The stewardship model

The stewardship model for state interventions, developed by the Nuffield Council on Bioethics, outlines ethical principles to guide government in fulfilling its responsibility to protect the health of vulnerable populations (19). Public policies to support the health of children with SHCN precede the stewardship model but are consistent with its ethical principles. Beginning in the 1930s, the federal government, working with states, assumed responsibility for the health of children with SHCN through policies to establish subspecialty clinics for "crippled" children. Policies have evolved to support children's access to health services that are comprehensive, coordinated, family-centered, and respectful of the family as a decision maker and partner with providers. This approach, although ideal for managing complex medical and behavioral conditions, is less suited for addressing health threats related to obesity; these require policies that support family- and community-based initiatives in addition to health services. Policies that help families and communities encourage children with SHCN to maintain healthy weights are consistent with the stewardship model.

### The dilemma of difference

The dilemma-of-difference construct (20) identifies policy concerns that are specific to children with SHCN and ethically might require different approaches. Under inclusive policies, children with SHCN and their families benefit from the multiple advantages associated with being "like all the other kids," but some may benefit more from policies that support specialized services. According to the dilemma of difference, the benefits of specialized services must be weighed against potential costs, including stigmatization, when children are labeled as different.

In addition to supporting their child's inclusion in mainstream activities, families often seek special services to support optimal outcomes despite the stigma that can accompany labeling a child with a specific diagnosis for eligibility and service provision purposes. Policies that support designated services and special accommodations for children with SHCN (eg, different educational materials) will be essential to enable certain children to reduce obesity risks. Families whose children have benefited from specialized services will respond well to policy-based efforts to prevent obesity, both inclusive and specialized. Families who seek specialized services, however, sometimes report roadblocks and disappointing outcomes — despite legal protections — that can influence their responsiveness to specialized obesity prevention approaches. Families facing disadvantages related to their race, ethnicity, or language when advocating for their children with SHCN may be discouraged from taking advantage of what is legally available. Obesity prevention initiatives for such families should acknowledge both their racial/ethnic or linguistic community and role as a parent of a child with SHCN. Determining the appropriate mix of inclusive and special obesity prevention initiatives to optimize benefits for children with SHCN may need to be decided at the level of the individual child and family.

Although the confluence of the policy domains of school-based obesity prevention and educational opportunities should support inclusion of children with special needs in school-based wellness initiatives, the extent to which these children are included is unknown. States' and communities' responsibility to educate children with special needs was established during the 1970s, first through federal court rulings and subsequently through passage of the Education for All Handicapped Children Act of 1975 (Public Law 94-142), presently enacted as the Individuals with Disabilities Education Act (IDEA) as amended in 2004 (Public Law 108-446) (21). Section 504 of the Rehabilitation Act of 1973 (22) and the Americans with Disabilities Act of 1990 (23) provide additional protections. As schools continue to serve as sites for obesity prevention, and wellness initiatives are integrated into curricula, anything less than the full participation of children with SHCN in these initiatives is unethical, and perhaps illegal, because their exclusion violates their rights to a free and appropriate public education. Parents can seek redress through the Individualized Education Plan process, the child-specific blueprint for education programming specified in IDEA, or section 504 accommodations, although doing so requires acknowledging the child's difference, and may result in the parents' being labeled difficult. Systemic change is required to create healthy school environments for all children, including children with SHCN.

Health care providers also monitor children's weights and counsel parents about the importance of healthy eating and physical activity, and obesity prevention is emerging as part of routine preventive health care for children (11). Approximately 91% of children with SHCN have health insurance (1) and are positioned to benefit from heightened health care attention to obesity prevention. The federal Patient Protection and Affordable Care Act, signed into law in 2010, will — if fully implemented — ensure that all children have health insurance and access to routine preventive health care (24). Although an American Academy of Pediatrics policy calls for physicians to screen all children annually for excess weight gain, no published data exist regarding the degree to which children with SHCN receive preventive screening (25). Because children with SHCN often see subspecialists rather than primary care providers, routine preventive services may not be consistently delivered (26). In a review of 51 proposed federal policy options for addressing childhood obesity, none focused on the health care system (27).

### Ethics and the role of families in policy making

We recognize the importance of autonomy versus paternalism in ethical discourse and families' long-standing formal role in health policy deliberations. Policy initiatives designed to promote the health of children with SHCN traditionally acknowledge the additional responsibilities and challenges their families face and often include provisions for extra information and support. Similar designated support for families will be essential in initiatives to prevent obesity among children with SHCN. Dietary and physical activity guidelines that read as if one size fits all will fail to provide families of children with SHCN with the resources they need to encourage their children to eat well and be physically active. Policy makers involved in the processes of developing, implementing, and evaluating policies for children with SHCN have learned the value of including families. From an ethical perspective, this approach respects the autonomy of families, in contrast to more paternalistic policy-making approaches. Although families believe that their involvement helps ensure usability and flexibility, broad-based obesity policy initiatives have rarely involved them. Given this history and federal laws and regulations requiring family involvement (eg, in policy making and oversight roles in state Title V programs [28]), many families of children with SHCN will expect and come to demand that policy responses to obesity for all children include both supports for families and provisions to include families formally in the policy formulation and implementation process. The stewardship model stresses the importance of consulting with people about policy measures that affect them and would support a policy-making role for families.

## Child Obesity Policy: Promises and Challenges

Data indicate that children with SHCN account for part of the increasing prevalence of childhood obesity in the general population. The federal government, in partnership with states, supports a network of services to promote the health of this vulnerable population through increased access to quality health services. Addressing new health risks associated with obesity requires policies that support family- and community-based initiatives in addition to health services. Ethical concerns about child obesity policy, however, may slow or prevent development of workable solutions (6).

We examined 4 ethical considerations that influence responses to child obesity policy from the perspective of children with SHCN. Our goal was to identify issues that are pertinent to efforts to optimize the benefits of child obesity policy for these children. The policy history of children with SHCN and their families is likely to result in support for broad-based multifactorial efforts to promote healthy weights in children. Still, children with SHCN will benefit from governmental policy solutions for obesity intended for the wider world of children if and only if they are guaranteed participation. Policy responses must also be sufficiently robust to address the needs of certain children and families who require more specialized initiatives. Calibrating the correct mix of inclusive and special obesity prevention initiatives and determining the strategies that ensure full participation in school and community activities present challenges.

Program models, methods, and materials to enable children with SHCN to participate fully in activities that promote healthy weights in their schools and communities are in short supply. Families of children with SHCN — collectively and individually — can help determine which policy responses most benefit their children and advocate for their inclusion; families have demonstrated their effectiveness in other policy domains. Policy makers should be aware of the growing racial/ethnic and linguistic diversity of the US population and the particular needs and concerns of families of children with SHCN from minority and low-income communities. Policy solutions designed to address the needs of all children, including children with SHCN, are required to create healthy environments for children and to ensure that all children have a healthy life.

## Figures and Tables

**Table 1:** Sociodemographic Characteristics of Children With Special Health Care Needs^a^

**Characteristic**	% of All Children
**Sex**	
Male	16.1
Female	11.6
**Age, y**	
0-5	8.8
6-11	16.0
12-17	16.8
**Family income, % of federal poverty level^b^**	
0-99	13.9
100-199	14.0
200-399	13.6
≥400	14.0
**Race/ethnicity**	
Mixed race	18.0
Non-Hispanic white	15.5
Non-Hispanic black	15.0
American Indian/Alaska Native	14.5
Hispanic, primary language English	13.0
Native Hawaiian/Pacific Islander	11.5
Asian	6.3
Hispanic, primary language Spanish	4.6

a Source: Health Resources and Services Administration (1). "Special health care needs" defined as the presence of a chronic physical, developmental, behavioral, or emotional condition and the need for health and related services that differ from those required by children generally in type and in intensity (7).

b 100% of the federal poverty level was $19,350 for family of 4 in 2005.

**Table 2:** Prevalence of Health-Related Characteristics Among Children With Special Health Care Needs^a^

**Characteristic**	% of Children With Special Health Care Needs
**Consequences of special health care needs**	
Child's use of or need for prescription medication	78.4
Elevated service use (medical care, mental health, education)	38.5
Emotional, behavioral, or developmental problem	28.4
Limitation in activities, compared with peers	21.3
Child's use of or need for special therapy (physical, occupational, speech)	17.5
**Functional difficulties, by type**	
Difficulty with any bodily function (eating, dressing)	57.4
Difficulty with participation in any activity (walking, running)	49.3
Emotional or behavioral difficulty	41.9
**Selected conditions**	
Allergies	53.0
Asthma	38.8
Attention deficit disorder/attention deficit hyperactivity disorder	29.8
Depression/anxiety/other emotional problem	21.1
Mental retardation	11.4
Autism or autism spectrum disorders	5.4
Seizure disorders	3.5

a Source: Health Resources and Services Administration (1). "Special health care needs" defined as the presence of a chronic physical, developmental, behavioral, or emotional condition and the need for health and related services that differ from those required by children generally in type and in intensity (7).
